# Design of an In Vivo‐Like Phantom for Quantitative Body CEST MRI

**DOI:** 10.1002/mrm.70585

**Published:** 2026-09-09

**Authors:** Petr Menshchikov, Petr Bulanov, Neele Kempa, Mark E. Ladd, Andreas Korzowski, Philip S. Boyd

**Affiliations:** ^1^ Division of Medical Physics in Radiology German Cancer Research Center (DKFZ) Heidelberg Germany; ^2^ German Cancer Consortium (DKTK), Core Center Heidelberg Heidelberg Germany; ^3^ Faculty of Physics and Astronomy, Heidelberg University Heidelberg Germany; ^4^ Faculty of Medicine, Heidelberg University Heidelberg Germany; ^5^ Department of Cardiology Pulmonology Diseases and Vascular Medicine, Medical Faculty, Heinrich‐Heine‐University Düsseldorf Germany

**Keywords:** body CEST MRI, BSA, CEST, fat, gelatin, phantom

## Abstract

**Purpose:**

To design and validate a reproducible in vivo‐like fat‐containing CEST phantom that enables robust quantitative CEST MRI for body applications by providing physiologically realistic relaxation properties, relevant endogenous CEST contrasts (amide, guanidino, rNOE, and semi‐solid MT), and a controlled fat component.

**Methods:**

An agar‐based water‐fat CEST phantom was designed using agar, gelatin, and bovine serum albumin, with adjustable fat fraction (FF) achieved through stable water‐fat emulsions. Phantom composition was tuned to reproduce tissue‐relevant T_1_ and T_2_ relaxation times and physiological pH. The accuracy and temporal stability of FFs and CEST contrasts were assessed over a 3‐month period. Low‐power CEST MRI at 7T was performed to quantify amide, guanidino, and rNOE contrasts using the inverse metric MTR_Rex_, and their concentration dependence and temporal stability were evaluated.

**Results:**

The proposed phantom‐generated Z‐spectra closely resembled in vivo body CEST measurements and reproduced major endogenous CEST and semi‐solid MT effects with controllable contrast levels. Stable and reproducible FF values were achieved for FFs up to 50% over the investigated 3‐month period, covering the clinically relevant range for most body tissues. Quantitative CEST metrics demonstrated linear concentration dependence and good temporal stability.

**Conclusion:**

An in vivo‐like, fat‐containing CEST phantom was developed and validated as a reproducible and physiologically relevant reference standard for quantitative body CEST MRI. By enabling controlled investigation of fat‐induced effects on CEST contrast quantification, this phantom supports the standardized validation of fat suppression and correction strategies and facilitates methodological development and cross‐study comparability in body CEST imaging.

## 1 Introduction

Chemical exchange saturation transfer (CEST) MRI provides endogenous contrast mechanisms sensitive to protein content, cellularity, pH, and mobile macromolecules [1, 2]. Several distinct CEST‐related signal contributions can be observed, including amide (APT), guanidino and amine proton exchange, semi‐solid magnetization transfer (ssMT), and exchange‐relayed nuclear Overhauser effects (rNOE). These contrasts have demonstrated substantial clinical value, particularly in neuroimaging [3] and neuro‐oncology applications [4, 5, 6, 7]. More recently, these contrasts have attracted increasing interest in body MRI, with emerging applications in the breast [8, 9], liver [10], and kidneys [11]. Although the available evidence remains limited in body CEST imaging, reported studies suggest that CEST signals are associated with clinically relevant tissue characteristics, including (i) histological features such as tumor grade [12] and proliferation [9], as well as (ii) metabolic and microenvironmental alterations, such as extracellular pH [13] and protein or glycogen content [14].

A fundamental challenge for body CEST MRI is the presence of adipose tissue. Fat‐related signals distort the Z‐spectrum [8, 15, 16] and therefore substantially bias quantitative CEST metrics, such as MTR_Rex_ [17], MTR_asym_ [18, 19], and so on [20]. Consequently, effective CEST‐specific fat artifact removal strategies are essential and must be rigorously validated. However, in vivo validation is inherently limited by physiological variability, spatially heterogenous fat distribution, and the lack of truly fat‐free reference regions in many body organs. Conversely, conventional aqueous solutions containing water‐soluble CEST agents (e.g., bovine serum albumin (BSA), creatine, or other metabolites) are inherently unable to reproduce lipid‐driven artifacts and therefore cannot be used to systematically evaluate the performance, robustness, and bias of fat‐related correction approaches in body CEST imaging. To address this gap, there is a clear need for a dedicated CEST phantom that combines physiologically realistic relaxation properties with relevant endogenous CEST contrasts and a controlled, reproducible fat component.

In this study, we present the design of an agar‐based water‐fat CEST phantom that reproduces physiological relaxation properties, major endogenous CEST effects (mediated by gelatin and BSA), and a customizable range of fat fractions (FF). We further demonstrated the temporal stability of its MR properties. Together, these features enable standardized and systematic evaluation of fat‐induced effects on CEST contrast quantification, supporting the use of this phantom as a reference standard for the development and validation of fat suppression and correction methods in body CEST MRI.

## 2 Methods

### 2.1 Design of Phantoms

In the current work, two phantoms were constructed, each consisting of four plastic vials (Figure 1A): (i) Phantom 1 with constant gelatin concentration and varying BSA concentration without fat (Figure 1B), and (ii) Phantom 2 with constant gelatin and BSA concentrations and incrementally increasing FF (Figure 1C). Both phantoms were filled with a water‐based solution (brown) containing 49% polyvinylpyrrolidone (PVP) and 1.8% NaCl, adjusted to mimic in vivo electrical properties, with a relative permittivity of 44 and an electrical conductivity of 0.56 S/m at 300 MHz [21].

**FIGURE 1:**
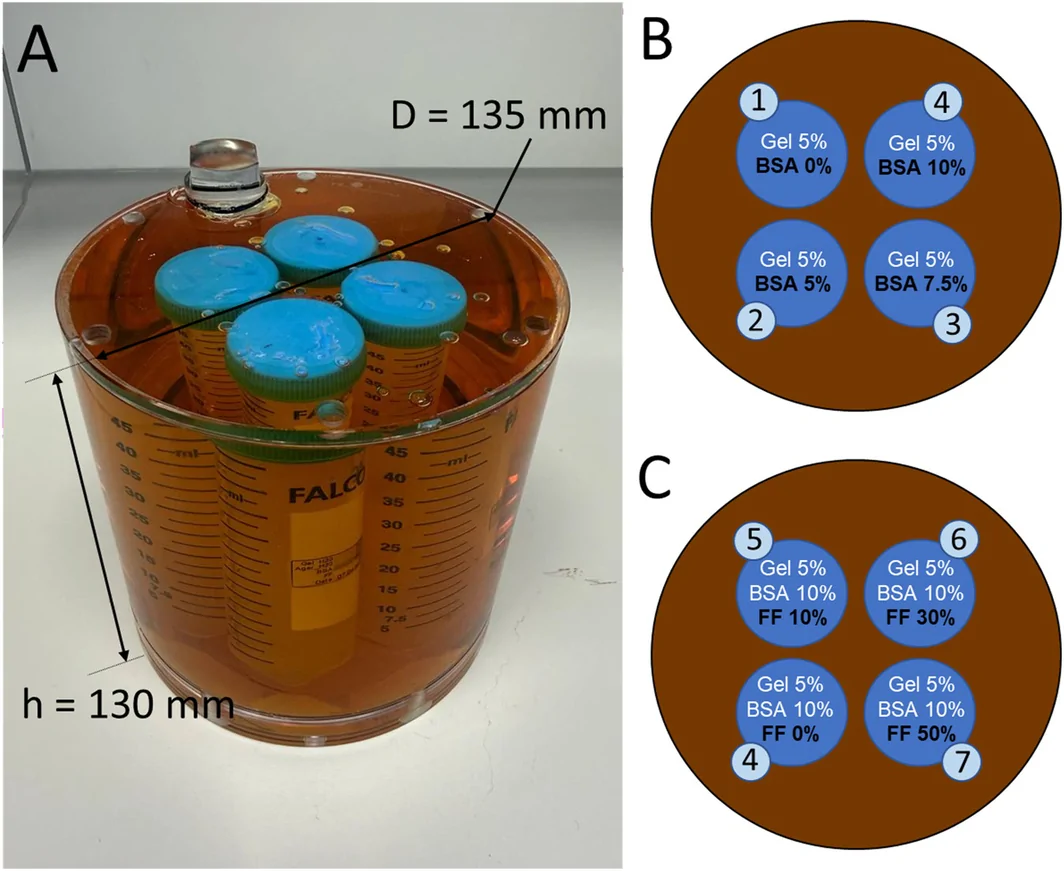
Overview of the experimental phantom design. The PMMA bottle with represented sizes is filled with a PVP‐based background solution electrically mimicking surrounding tissue (A). Schematic representation of Phantom 1, consisting of four vials with constant gelatin concentration (5%) and varying BSA concentration (0%–10%) without fat (B). Schematic representation of Phantom 2, consisting of four vials with constant gelatin (5%) and BSA (10%) concentrations and incrementally increasing fat fraction (FF = 0%–50%). Numbers (1–7) denote individual vials corresponding to the compositions listed in Table 1. Polyvinylpyrrolidone (PVP); Bovine serum albumin (BSA); gelatin (Gel); fat fraction (FF).

Each vial was prepared individually according to the material proportions listed in Table 1, following the same fabrication procedure established in this study (Figure 2) and precisely described in the “Individual Phantom Production” paragraph. The concentrations of preparation materials listed in Table 1 were selected to (i) span physiologically relevant variations of the FF and CEST contrasts and (ii) maximize the available FF (vial #7, FF = 50%), while preserving structural integrity and reproducible MRI properties.

**TABLE 1:** Phantom vial composition, concentrations of preparation materials and relaxation parameters as measured at 7T. Measured values are reported as mean ± standard deviation.

Tube number			1	2	3	4	5	6	7
Phantom			1			1 and 2	2		
Basic information		BSA, %wt/wt	0.0	5.0	7.5	10.0	10.0	10.0	10.0
		Gelatin, %wt/wt	5.0	5.0	5.0	5.0	5.0	5.0	5.0
		Agar, %wt/wt	2.2	2.2	2.2	2.2	2.2	2.2	2.2
		FF, %wt/wt	0	0	0	0	10	30	50
Concentrations of preparation materials	Stock water solution (SWS)	Water (pH 7.3) + gad, mL	92.9	77.5	69.6	61.6	61.6	61.6	61.6
		Sodium bensoate, g	0.20	0.20	0.20	0.20	0.20	0.20	0.20
		Tween 80, mL	1.30	1.30	1.30	1.30	1.30	1.30	1.30
	Agar solution	SWS, mL	66.4	55.3	49.7	44.0	44.0	44.0	44.0
		Agar, g	2.15	2.15	2.15	2.15	2.15	2.15	2.15
	Gelatin solution	SWS, mL	26.5	22.1	19.9	17.60	17.60	17.60	17.60
		Gelatine, g	4.9	4.9	4.9	4.9	4.9	4.9	4.9
	BSA solution	BSA 30% wt/wt (pH 7.3), mL	0.0	15.5	23.4	31.4	31.4	31.4	31.4
	Oil solution	Peanut oil, mL	n/a	n/a	n/a	n/a	12.0	47.0	111.0
		Span 80, mL	n/a	n/a	n/a	n/a	0.2	0.9	2.2
T_2_ and T_1_ values (ms)		T_2_ water (sLASER), ms	92.4 ± 1.2	70.6 ± 0.9	65.4 ± 0.9	60.1 ± 0.8	19.9 ± 0.5	15.2 ± 0.4	< 7–10
		T_2_ fat (sLASER), ms	n/a	n/a	n/a	n/a	40.5 ± 2.4	40.9 ± 2.7	36.2 ± 2.2
		T_1_ water (IR GRE), ms	1593 ± 10	1495 ± 21	1443 ± 11	1394 ± 16	1414 ± 11	1417 ± 20	1378 ± 35

*Note:* The final gadolinium concentration in the phantom tubes was approximately 0.93 μM.

Abbreviations: BSA, bovine serum albumin; FF, fat fraction; wt/wt, weight/weight; IR‐GRE, inversion recovery gradient echo; sLASER, semi‐localized by adiabatic selective refocusing; SWS, stock water solution; n/a, not applicable.

**FIGURE 2:**
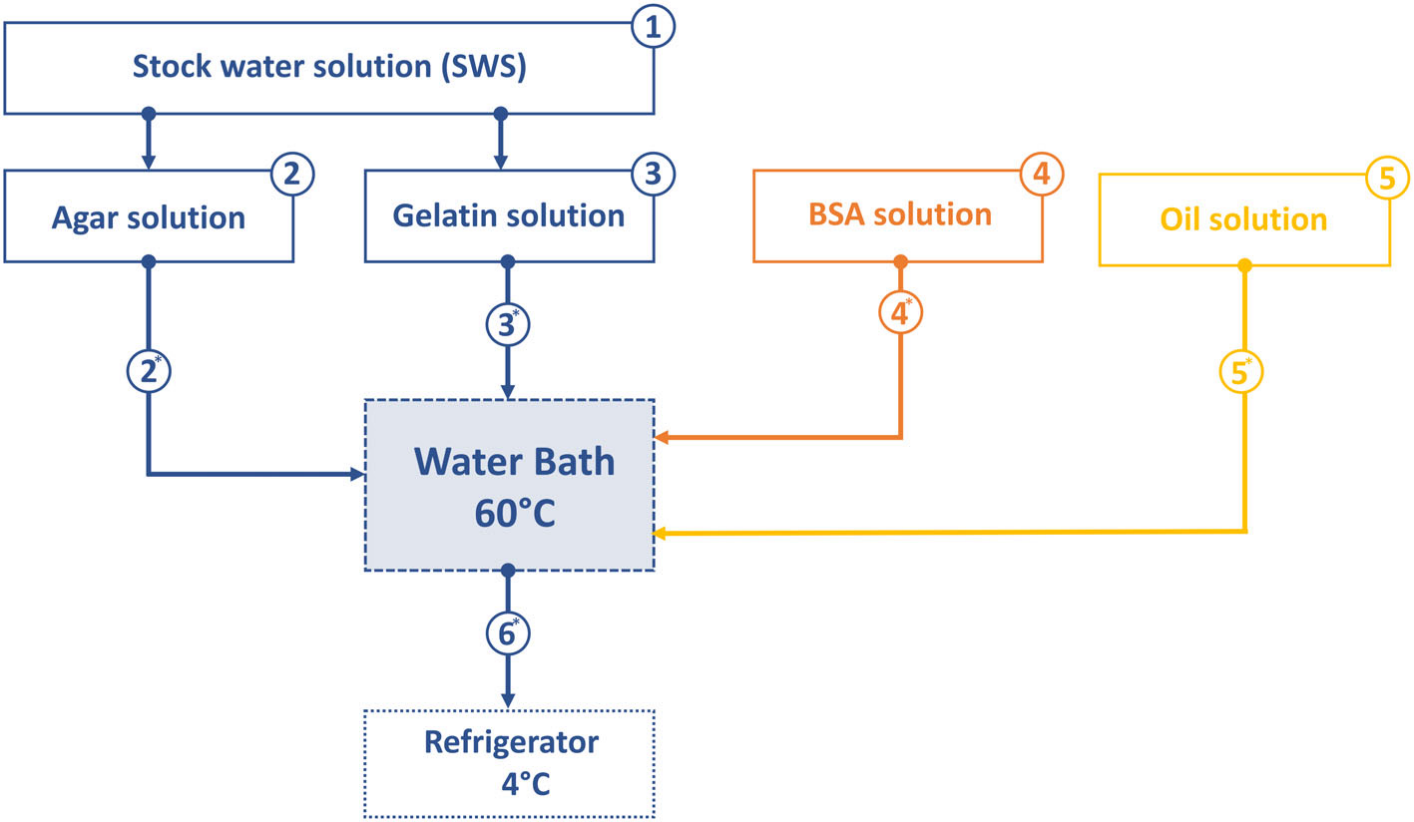
General scheme of the individual phantom vial production. Step 1: Preparation of the stock water solution (SWS) containing sodium benzoate and Tween 80 in a gadolinium‐doped buffer, followed by distribution into portions for agar and gelatin solutions. Step 2–2*: Preparation of the agar solution by bringing the agar‐SWS mixture to boil and subsequent cooling to 60°C under continuous stirring. Step 3–3*: Preparation of the gelatin solution by hydration and dissolution of gelatin in SWS and slow incorporation into the agar solution under continuous stirring. Step 4–4*: Preparation of the BSA solution (30% wt/wt, pH 7.3), heating to 60°C, and slow injection into the agar‐gelatin mixture under stirring. Step 5–5*: Preparation of the oil solution using peanut oil and Span 80, followed by controlled emulsification into the agar‐gelatin‐BSA mixture. Step 6*: Filling of the final mixture into vials, cooling at 4°C for solidification.

### 2.2 Individual Phantom Production

#### 2.2.1 Step 0. Buffer and Gadolinium Solution Preparation

Phantom fabrication starts with the preparation of a distilled water solution with adjusted pH = 7.3 using a phosphate buffer. The buffer is supplemented with the 0.5 M gadolinium‐based contrast agent (20 μL per 1 L; MultiHance, Bracco Imaging Deutschland GmbH) to modify the relaxation properties. To ensure the consistency of the phantom composition, the same gadolinium‐doped buffer solution was used for all vials.

#### 2.2.2 Step 1: Stock Water Solution (SWS) Preparation

First, sodium benzoate (Sigma‐Aldrich, catalog no. 71300) and a water‐soluble surfactant TWEEN 80 (Sigma‐Aldrich, Saint Louis, MO, USA; catalog no. P1754) are dissolved in a gadolinium‐doped buffer under magnetic stirring using a hotplate set to 150°C (Step 1, Figure 2). Tween 80 is slowly added to the buffer using a syringe, with the needle positioned a few millimeters above the center of the solution [22]. The resulting SWS is then divided into two portions for the preparation of the agar and gelatin solutions.

#### 2.2.3 Step 2: Agar Solution Preparation

Agar powder (Merck KGaA, Darmstadt, Germany; catalog no. 05039) is gradually added to the SWS under continuous stirring. The mixture is then brought to a boil on a hotplate (hotplate temperature set to 350°C), following standard agar handling procedures [22] while stirring until the solution becomes clear and homogeneous. Afterwards, the glass container with the aqueous agar solution is transferred to a water bath and cooled to 60°C under continuous stirring (Step 2*, Figure 2). The water bath temperature is maintained at 60°C prior to protein incorporation to minimize the risk of thermal denaturation and aggregation of BSA, which becomes conformationally unstable above this temperature, with irreversible structural changes occurring at higher temperatures [23]. In addition, this temperature corresponds to the dissolution range of gelatin, ensuring complete solubilization while maintaining consistent physicochemical conditions across samples and preserving reproducible CEST properties.

#### 2.2.4 Step 3: Gelatin Solution Preparation

Gelatin powder (Merck KGaA, Darmstadt, Germany; catalog no. 104070) is mixed with the SWS (Table 1) chilled to 40°C. The mixture is then placed in a refrigerator at 4°C for 10–15 min to allow the gelatin to hydrate and swell, forming a thick mass. Subsequently, the beaker containing the bloomed gelatin is transferred to a water bath at 60°C without stirring until the gelatin is fully dissolved and the solution becomes transparent. Any foam that forms on the surface of the solution is carefully removed. Finally, the gelatin solution is slowly poured into the agar solution under continuous low‐speed stirring to ensure homogeneous mixing (Step 3*, Figure 2).

#### 2.2.5 Step 4: Preparation of the BSA Solution

BSA powder (heat shock fraction, pH 7, Sigma‐Aldrich, catalog no. A9647) is slowly mixed with the SWS buffered to pH 7.3 to obtain a final BSA concentration of 30% wt/wt. Alternatively, commercially prepared BSA solutions may be used; however, particular care should be taken to ensure pH matching, as deviations can affect the final phantom pH. The BSA solution is then transferred to a water bath at 60°C. The heated BSA is slowly injected into the agar‐gelatin mixture using a syringe and under continuous low‐speed stirring to ensure homogeneous incorporation (Step 4*, Figure 2).

#### 2.2.6 Step 5: Preparation of the Oil Solution

An oil‐soluble surfactant Span 80 (Sigma‐Aldrich, Saint Louis, MO, USA; catalog no. 840123) is mixed with peanut oil preheated to 60°C. Peanut oil is selected as the lipid component due to its well‐characterized proton MR spectrum, which closely resembles that of human adipose tissue [24]. Span 80 is slowly added using a syringe, following the same procedure as for Tween 80, to ensure homogeneous dispersion. The prepared oil solution is mixed into the agar‐gelatin‐BSA mixture under continuous stirring to form a homogeneous emulsion.

#### 2.2.7 Step 6: Vial Preparation and pH Measurement

The final mixture is poured into 50 mL plastic containers and placed in a refrigerator at 4°C for at least 2 h until completely solidified. Residual emulsion samples are reheated and centrifuged to separate the aqueous phase, which is then collected and cooled to 20°C. pH is measured from the aqueous phase using pH test strips (Reflectoquant, Merck KGaA, Darmstadt, Germany) and quantified with a reflectometer (RQflex 20, Merck KGaA, Darmstadt, Germany), providing a resolution of 0.1 pH units.

### 2.3 MRI Acquisition and Post‐Processing

CEST and ^1^H MRS measurements were performed on a 7T MAGNETOM whole‐body MR scanner (Siemens Healthineers, Erlangen, Germany) using a 64‐channel receive‐only head coil (Rapid Biomedical GmbH, Rimpar, Germany). The phantom was stored in a refrigerator at 4°C throughout all intervals between scans. Prior to each measurement, the phantom was placed in the scanner room for 24 h to equilibrate to room temperature (∼20°C).

The MRI protocol consisted of: (i) two CEST scans acquired at different B_1,pa_ (power amplitude) saturation amplitudes (B_1,pa_ = 0.6 and 0.9 μT [25]; 297 Gaussian pulses, t_p_ = 15 ms; DC = 80%, T_rec_/T_rec_M0_ = 2/12 s) at 98 frequency offsets unequally sampled between ± 300 ppm (Supplementary A in Supporting Information); (ii) B_0_/B_1_ mapping by means of a fat‐suppressed WASABI [26] saturation (B_1_ = 7 μT, single rectangular pulse, t_p_ = 2.5 ms, t_rec_ = 5.6 s, equally sampled frequency offsets between ± 4 ppm) (iii) fat‐suppressed inversion recovery T_1_ mapping (Supplementary A in Supporting Information). All images used the same readout: a centric‐reordered 2D GRE readout (TE/TR = 2.04/4.20 ms; FOV, 196 × 196 mm^2^; matrix 128 × 128; slice thickness = 5 mm; bandwidth = 1220 Hz/pixel) [27]. The imaging slice was positioned orthogonally to the tubes through their center.

CEST data processing was performed in MATLAB R2019b (MathWorks, Natick, MA, USA) using in‐house code of a well‐established CEST evaluation pipeline [4], which included the following steps: (i) normalization; (ii) B_0_ and B_1_ correction [28]; (iii) fitting of the Z‐spectra by means of a 5‐pool (amide, guanidino, ssMT, rNOE and water) Lorentzian fit model. As a result, the isolated rNOE (Δω_rNOE_ ≈ −3.5 ppm), amide (Δω_amide_ ≈ 3.5 ppm), and guanidino (Δω_gua_ ≈ 2.0 ppm) contrasts, were calculated by means of the inverse metric (i.e., the relaxation‐compensated magnetization transfer ratio, MTR_Rex_ [17]).

T_2_ relaxation times of fat (methylene, —CH_2_— peak at ∼−3.4 ppm) and water resonances were additionally measured for each vial using single‐voxel sLASER spectroscopy (TE from 68 to 138 ms with 10 ms increment; TR = 10 s; VOI size = 10 × 10 × 20 mm^3^). 1H MRS Spectra were analyzed using the AMARES [29] algorithm implemented in the jMRUI software.

FF mapping was performed on a 3T MAGNETOM Vida MR scanner (Siemens, Erlangen, Germany) equipped with a 64‐channel receive head/neck coil using a 3D Dixon VIBE sequence (3D Dixon VIBE; TE = 1.05/2.46/3.69/4.92/6.15/7.38 ms; TR = 9.0 ms; FOV, 380 × 333 mm^2^; matrix 160 × 111; interpolated pixel size = 1.2 × 1.2 mm^2^, slice thickness = 3.5 mm (64 slices); acceleration Factor 4; bandwidth = 1080 Hz/pixel). FF maps were reconstructed directly on the MRI console.

The stability of the phantom vials was assessed by visual inspection of the emulsions to identify phase separation. In addition, the stability of MTR_Rex_ and FF values measured in ROIs drawn in the central part of the tubes was evaluated using repeated measurements. Specifically, four measurements were performed: the first acquisition was conducted immediately after phantom fabrication, followed by repeated measurements at 1, 2, and 3 months after that.

## 3 Results

The phantoms were successfully fabricated according to the proposed procedure, yielding stable emulsions across the targeted range of compositions. Overall, visual inspection during a 3‐month observation period revealed only for vial #7, corresponding to the highest fat content (FF = 50%), a small amount of oil separation. This was observed after approximately 2 weeks, forming a superficial oil layer.

Measured T_1_ and T_2_ relaxation times are summarized in Table 1. Both T_1_ and T_2_ relaxation times of water decreased with increasing BSA concentration. In addition, a significant decrease in T_2,water_ was observed with increasing FF, whereas the T_2,fat_ of the main fat resonance remained largely unchanged across different FF levels. Measured pH values in the vials were 7.2–7.3, consistent with the pH of the SWS and BSA solutions, both buffered to pH 7.3.

The resulting averaged Z‐spectra acquired at 7T obtained from vials 1–4 of phantom 1 (fat‐free) with varying BSA concentrations are shown in Figure 3A. In the absence of BSA, the dominant CEST‐related contributions in the Z‐spectrum originate from the gelatin, including a broad MT signal, spectrally distinct amide, guanidino, and rNOE signals (Figure S1). With increasing BSA concentration, a pronounced increase in the rNOE signal is observed (Figure 3B). At the same time, the amide and guanidino signals become stronger and broader.

**FIGURE 3:**
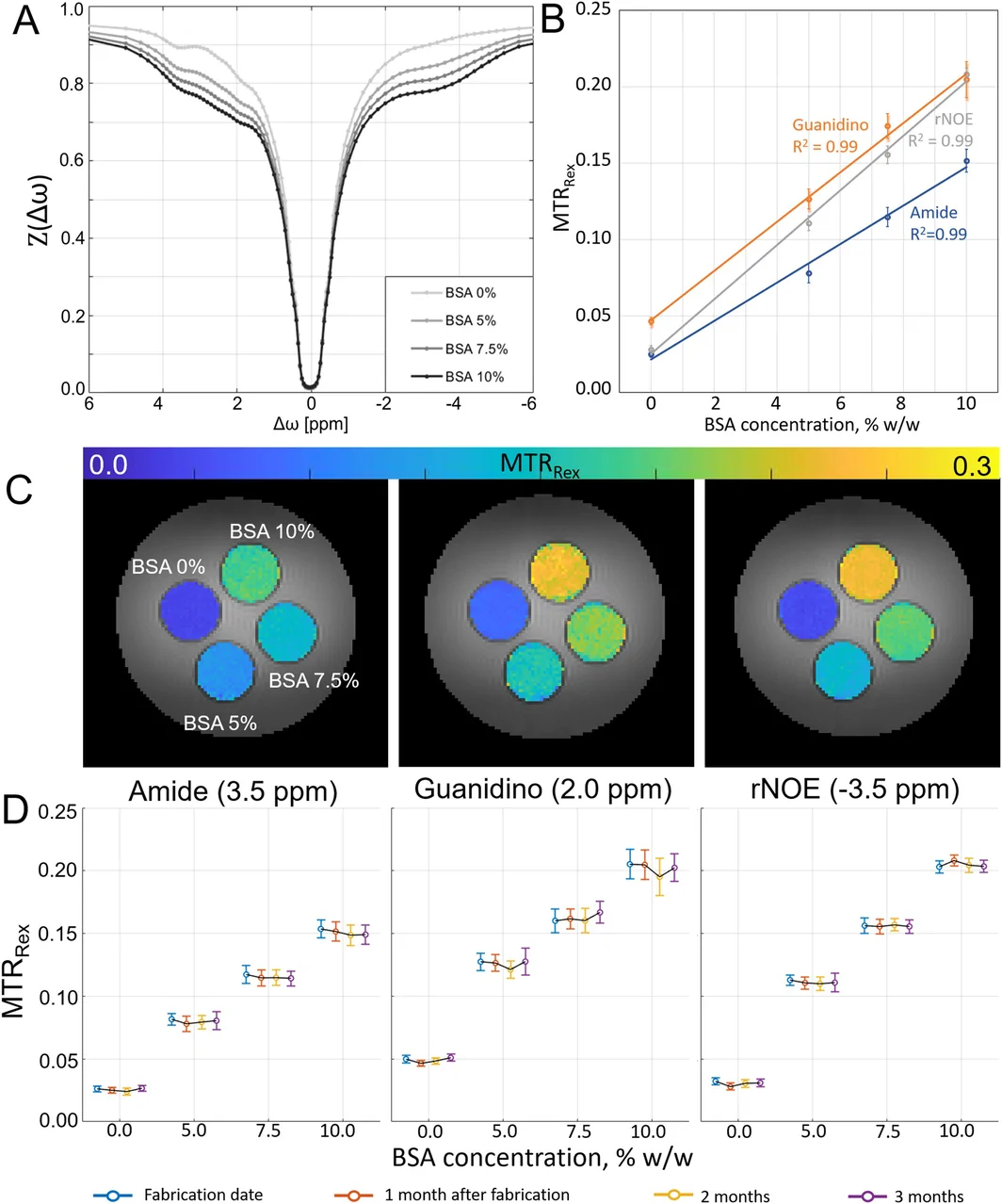
CEST imaging results obtained in the fat‐free phantom (phantom 1) at 7T. (A) Representative averaged B_0_‐ and B_1_‐corrected Z‐spectra (B_1_ adjusted to 0.7 μT) acquired from vials 1–4 containing 5% (wt/wt) gelatin with increasing bovine serum albumin (BSA) concentrations (cf Figure 1B). (B) Dependence of measured and B_1_‐corrected MTR_Rex_ values for amide (3.5 ppm), guanidino (2.0 ppm), and rNOE (−3.5 ppm) contrasts on BSA concentration (wt/wt). (C) Representative MTR_Rex_ maps for amide, guanidino, and rNOE contrasts. (D) Temporal evolution of MTR_Rex_ values (mean ± standard deviation) for amide, guanidino, and rNOE contrasts in each vial, measured immediately after phantom fabrication and after 1, 2, and 3 months of storage.

MTR_Rex_ maps presented in Figure 3C appear spatially homogeneous inside the vials. Quantitative analysis shows that MTR_Rex_ values for the amide, guanidino, and rNOE contrasts increase linearly with BSA concentration, demonstrating strong correlations (Figure 3B), and remain stable over a 3‐month observation period (Figure 3D).

The resulting averaged Z‐spectra obtained from vials 4–7 of phantom 2 with varying FF are shown in Figure 4A. With increasing FF, pronounced fat‐related artifacts are observed in the Z‐spectrum, including: (i) an apparent reduction of the direct water saturation (DWS) peak around 0 ppm caused by the contribution of fat signal that is not saturated at this frequency offset; and (ii) a strong lipid signal in the range of approximately −2 to −4 ppm, spectrally overlapping with the rNOE signal. Z‐spectra after fat correction using the fat‐corrected normalization proposed by Zimmerman et al. are shown in Figure S3. It should be noted that the Z‐spectra were acquired at an in‐phase condition of water and fat signals (TE = 2.04 ms at 7T); and the appearance and relative contribution of these lipid‐related artifacts would be substantially different for other TEs.

**FIGURE 4:**
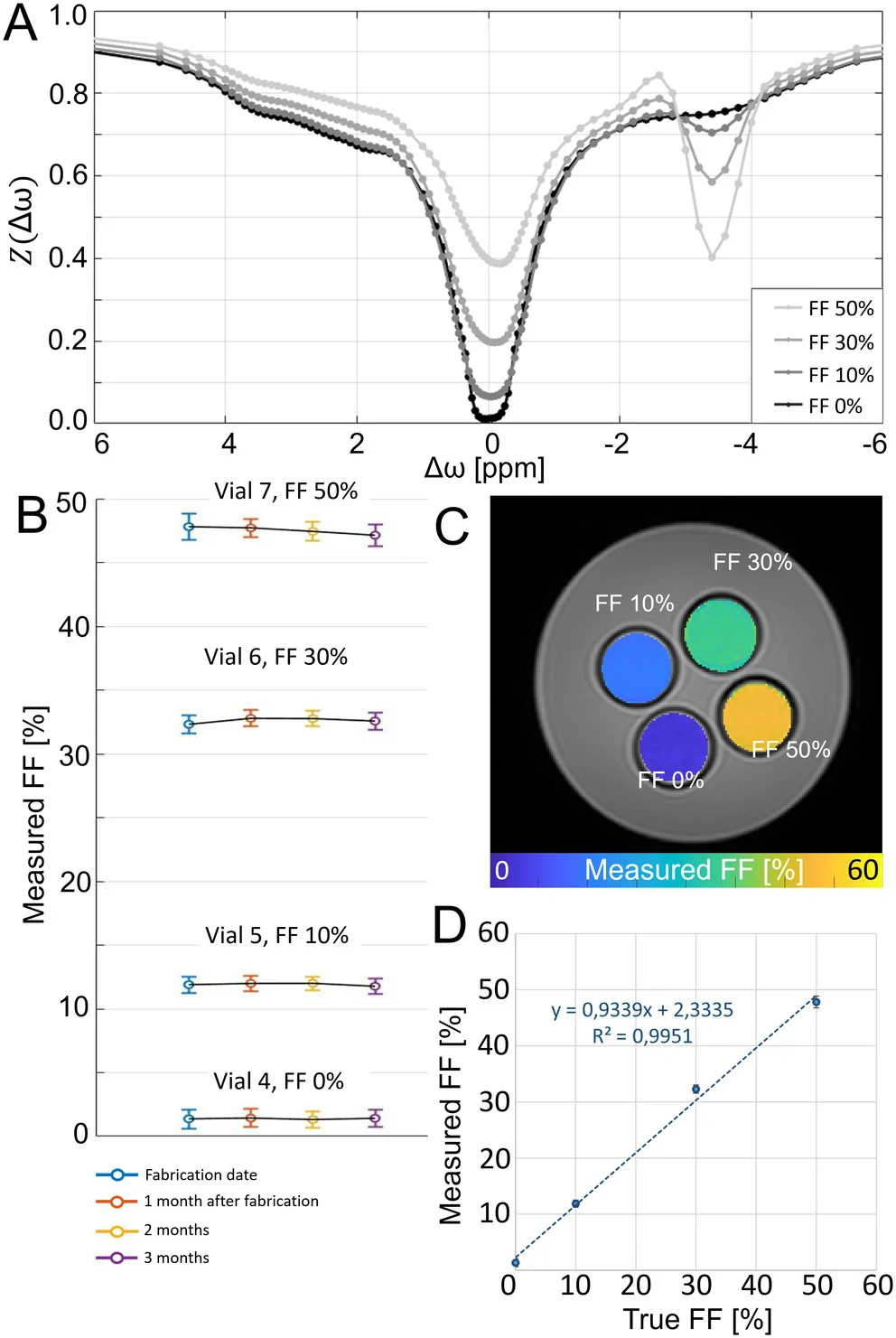
CEST and FF imaging results obtained in the fat‐containing phantom (phantom 2). (A) Representative Z‐spectra averaged across ROIs. B_0_‐ and B_1_‐corrected Z‐spectra (B_1_ adjusted to 0.7 μT) acquired from vials 4–7 containing 5% (wt/wt) gelatin, 10% (wt/wt) bovine serum albumin (BSA), and increasing fat fraction (FF) (cf Figure 1C). (B) Temporal evolution of FF values (mean ± standard deviation) for each vial, measured immediately after phantom fabrication and after 1, 2, and 3 months of storage. (C) Representative FF map. (D) Scatterplot of measured FF values (immediately after phantom fabrication) as a function of nominal FF.

A representative FF map is shown in Figure 4C and appears spatially homogeneous inside each vial. The measured FF values show strong agreement with the nominal FF values (Figure 4D). In addition, FF values measured at the vial centers remain stable over a 3‐month observation period, as illustrated in Figure 4B.

## 4 Discussion and Conclusions

This work introduces an efficient fabrication workflow for the generation of fatty‐tissue‐like CEST phantoms that does not require specialized or expensive equipment. Importantly, the proposed phantom demonstrates good temporal stability of both FF and CEST‐derived metrics over the investigated observation period (3 months), supporting its suitability for longitudinal and comparative studies. The resulting Z‐spectra closely resemble those observed in vivo, reflecting the careful design of the phantom to reproduce key tissue‐relevant CEST and MR properties.

The observed in vivo‐like behavior can be attributed to several factors: (i) Physiologically realistic T_1_ and T_2_ relaxation properties (Table ST_1_). T_1_ was primarily adjusted via the gadolinium concentration, whereas BSA and gelatin provided substantially smaller contributions. T_2_ relaxation times were controlled mainly by the agar content, as agar shortens T_2_ primarily by restricting water mobility within the gel network and increasing proton‐proton dipolar interactions, thereby accelerating transverse dephasing [30]. In addition, T_2_ decreased with increasing BSA concentration, most likely due to dipole–dipole interactions modulated by the overall molecular tumbling of BSA molecules, together with water exchange between the different molecular environments of the BSA [27, 31]. (ii) Combined usage of gelatin [32] and BSA [33] enabled CEST signal linewidths comparable to in vivo conditions. As already mentioned, BSA has broader amide and guanidino signals than gelatin, which can be attributed to the large molecular weight of the protein, that is, slow molecular tumbling. (iii) The phantom pH was fixed within the physiological pH range typical for healthy tissues and organs (7.2–7.4, Table S1). Together, these features allow the generation of Z‐spectra that closely match those encountered in vivo. Moreover, the proposed fabrication pipeline further allows for flexible adjustment of FF and CEST agent concentrations, providing a controlled and reproducible framework for systematic evaluation of robustness, operating limits, and comparability of different fat suppression and correction strategies, as well as their impact on quantitative CEST metrics. This is particularly important for body CEST MRI.

A weak rNOE‐like contribution was observed even without BSA. Since the 0% BSA vial still contained gelatin, this contribution may originate from the gelatin matrix, which is a collagen‐derived protein‐containing material containing macromolecular and aliphatic proton groups that could contribute to rNOE‐like effects. This is consistent with previous reports of gelatin‐related CEST effects, including amide and guanidino‐like contributions [34]. Additional study of CEST spectra with various gelatine concentrations (Figure S4) also confirms the presence of a small rNOE contribution, which was only possible to detect with the low power saturation regime used in our study, in contrast to work from Liang et al. [34].

Beyond fat‐related investigations, the proposed phantom is also well‐suited for conventional fat‐free CEST studies. By varying BSA [33] and gelatin [34] relative concentrations, different levels of amide, guanidino, MT, and rNOE contrasts can be modeled in a controlled manner. At 3T representative Z‐spectra exhibit broader spectral lines (including DWS), while the rNOE‐ and amide‐related signals remain visible, which is similar for in vivo. However, due to the reduced spectral resolution at 3T and wide DWS signal, reliable separation of the guanidino and amide contributions is not possible (Figure S2). Additional CEST‐signal‐generating compounds, such as creatine, arginine, glutamate, and glucose, and so on might potentially be incorporated into the phantom design to extend its applicability to other molecular imaging studies, but in this case, stability of the phantom and CEST contrasts must be precisely explored. The final pH is assumed to remain stable due to the identical buffering conditions of all aqueous components. The pH of the phantom can also be independently controlled by adjusting the pH of the BSA and SWSs, as the final phantom pH closely follows the pH of these components, as demonstrated in the present study. Longitudinal pH measurements were not performed in the present study. However, no changes in the Z‐spectra or the corresponding MTR_Rex_ maps were observed over the 3‐month observation period that would suggest substantial pH variations under the applied storage conditions.

A primary limitation of the proposed phantom is the maximum achievable FF. Stable emulsions without visible oil separation were reliably achieved up to about 50%, while higher FF values were increasingly unstable. Conventional water‐fat MRI phantoms often employ higher agar concentrations (≥ 3%) to stabilize emulsions with very high FF (≈75%) [22]. However, in the present design, maximum agar concentrations in the stock agar solution must remain below approximately 5% to avoid premature gelification and the corresponding rapid increase of viscosity (even at water bath temperatures of approximately 60°C), which impairs mixing efficiency and promotes air bubble formation as well as spatial inhomogeneity. Importantly, this limitation does not substantially reduce the practical relevance of the phantom, as most tissues of interest in body CEST MRI (including mammary gland [35] and liver, even in moderate to advanced steatosis [36], and other tissues (Table S1)), typically exhibit FF well below 40%. Therefore, the achievable FF range adequately covers the clinically relevant regime for evaluating fat‐related artifacts and fat suppression performance. Nevertheless, even at low FF values, storage of the phantom outside the refrigerator resulted in degradation of the gel structure, separation of the aqueous matrix and oil phase, and potentially accelerated growth of mold and bacteria in the water‐based gel, and therefore results in non‐reproducible results.

A second limitation concerns differences in water T_2_ relaxation times, especially between fat‐free and fat‐containing vials. In water‐fat emulsions, apparent water T_2_ is reduced due to a combination of enhanced interactions of water with the macromolecular agarose matrix [37] and additional transverse dephasing caused by diffusion of water molecules through microscopic field gradients arising from susceptibility differences between lipid droplets and the surrounding aqueous phase [38]. This effect may broaden the DWS peak in fat‐containing samples compared to fat‐free references, potentially complicating direct Z‐spectra comparison after fat suppression. However, the impact of these effects can be mitigated by analysis approaches that explicitly model individual spectral components, such as multi‐pool Lorentzian fitting along with inverse model‐based metrics including MTR_Rex_, or by employing asymmetry‐based metrics that are less sensitive to moderate changes in DWS linewidth. On the other hand, depending on the intended application, T_2_ values can be adjusted independently in fat‐free and fat‐containing vials using paramagnetic dopants, for example, MnCl_2_, CuSO_4_, and so on [39].

In conclusion, we developed an in vivo‐like, fat‐containing CEST phantom that provides a reproducible and physiologically relevant reference standard for robust quantitative body CEST MRI. By enabling controlled variation of fat content, relaxation properties, and endogenous CEST contrasts, the proposed phantom addresses an unmet critical need for standardized evaluation and validation of fat suppression and correction methods. This approach supports systematic assessment of fat‐related biases in quantitative CEST metrics and facilitates methodological development and cross‐study comparability in body CEST imaging.

## Funding

This work was supported by the Helmholtz Initiative and Networking Fund as part of the project “MIB2 – Molecular MR Imaging of the Brain and the Body” and by the German Cancer Consortium (DKTK) Seed Funding 2025 – 2027 as part of the project “MAMMACARE – Towards Molecular MR Mammography for Cancer Care”.

## Conflicts of Interest

The authors declare no conflicts of interest.

## Supporting information


**Figure S1:** Example of multi‐pool fitting of B_0_‐ and B_1_‐corrected Z‐spectra (B_1_ adjusted to 0.7 μT) acquired in the absence of fat (Phantom 1). The spectra are shown for vials containing increasing BSA concentrations (0%–10% wt/wt) at a constant gelatin content (5% wt/wt). The fitting model included contributions from rNOE, amide, guanidino, and semisolid MT pools, which are expected to contribute to the Z‐spectra of BSA [1] and gelatin [2] in the low‐power saturation regime [3,4]. This saturation regime was intentionally selected to preserve sensitivity to the BSA‐related rNOE signal while also capturing amide‐ and guanidino‐related contributions from the gelatin and BSA together.
**Figure S2:** CEST imaging results obtained in the fat‐free phantom (phantom 1) at 3 T. (A) Representative averaged B_0_‐ and B_1_‐corrected Z‐spectra (B_1_ adjusted to 0.7 μT) acquired from vials 1–4 containing 5% (wt/wt) gelatin with increasing bovine serum albumin (BSA) concentrations. Compared with the corresponding Z‐spectra acquired at 7 T, the Z‐spectra acquired at 3 T exhibit broader linewidths, which impede reliable separation of the amide and guanidino signals. In addition, spillover dilution due to the direct water saturation effects is also more pronounced at 3 T. (B) Dependence of measured and B_1_‐corrected MTR_Rex_ values for amide (3.5 ppm) and rNOE (−3.5 ppm) contrasts on BSA concentration (wt/wt). (C) Representative MTR_Rex_ maps for amide, guanidino, and rNOE contrasts.
**Figure S3:** CEST Z‐spectra obtained in the fat‐containing phantom (phantom 2) with and without fat‐corrected normalization. (A) Representative averaged across ROIs. B_0_‐ and B_1_‐corrected Z‐spectra (B_1_ adjusted to 0.7 μT) acquired from vials 4–7 containing 5% (wt/wt) gelatin, 10% (wt/wt) bovine serum albumin (BSA), and increasing fat fraction (FF). (B) Representative fat‐signal‐corrected and B_0_‐ and B_1_‐corrected Z‐spectra (B_1_ adjusted to 0.7 μT) with fat‐corrected normalization applied as described in Zimmermann et al. [5] averaged across ROIs. Fat‐corrected normalization enabled effective correction of the downfield part of the Z‐spectrum (amide and guanidino signals) for FF values up to 35%, as previously described in [5]. This behavior is clearly reproduced in the phantom. For vials 5 and 6 (FF = 10% and 30%), the corrected direct water saturation (DWS) region around 0 ppm and the downfield part of the Z‐spectrum (amide and guanidino signals) agree well both with each other and with the reference spectrum. In contrast, for the vial with FF = 50%, overcorrection of the downfield Z‐spectrum is observed.
**Figure S4:** CEST Z‐spectra obtained in the phantom with different gelatin concentrations. (A) Representative average across vials' ROIs. B_0_‐ and B_1_‐corrected Z‐spectra (B_1_ adjusted to 0.7 μT) acquired from vials containing 2.2% (wt/wt) and increasing gelatin concentration. A small rNOE contribution can be seen, which was only possible to detect with the low‐power saturation regime used in the current study. (B) Dependence of measured and B_1_‐corrected MTR_Rex_ values for amide (3.5 ppm), guanidino (2.0 ppm), and rNOE (−3.5 ppm) contrasts on gelatin concentration (wt/wt), which also confirms the presence of a small rNOE signal.

## Data Availability

The data that support the findings of this study are available on request from the corresponding author. The data are not publicly available due to privacy or ethical restrictions.
