# Supplementary material for: Design of an In Vivo‐Like Phantom for Quantitative Body CEST MRI
Source: Magn Reson Med. 2026 Sep 9;97(1):454–63. doi: 10.1002/mrm.70585 (PMC13746073; doi:10.1002/mrm.70585)
Supplement: Supplementary file 1 [file MRM-97-454-s001.docx]

**Supporting Information**

**Design of an In-Vivo-Like Phantom for Quantitative Body CEST MRI**

*Petr Menshchikov, Petr Bulanov, Neele Kempa, Mark E. Ladd, Andreas Korzowski, and Philip S. Boyd*

**Supplementary A**

**IR times [ms]**

20000, 16000, 14000, 12000, 10000, 9000, 8000, 7000, 6500, 6000, 5500, 5000, 4500, 4000, 3800, 3600, 3400, 3200, 3000, 2800, 2600, 2400, 2300, 2200, 2100, 2000, 1900, 1800, 1700, 1600, 1500, 1400, 1300, 1200, 1100, 1000, 900, 800, 700, 600, 500, 400, 300, 200, 100

**CEST offset list [ppm]**

300, 150, 75, 25, 8, 5, 4.6, 4.4, 4.2, 4, 3.9, 3.8, 3.7, 3.6, 3.5, 3.4, 3.3, 3.2, 3.1, 3, 2.9, 2.8, 2.7, 2.6, 2.5, 2.4, 2.3, 2.2, 2.1, 2, 1.9, 1.8, 1.7, 1.6, 1.5, 1.4, 1.3, 1.2, 1, 0.9, 0.8, 0.7, 0.6, 0.55, 0.5, 0.45, 0.4, 0.35, 0.3, 0.25, 0.2, 0.15, 0.1, 0.05, 0, -0.05, -0.1, -0.15, -0.2, -0.25, -0.3, -0.35, -0.4, -0.45, -0.5, -0.55, -0.6, -0.7, -0.8, -0.9, -1, -1.2, -1.4, -1.6, -1.8, -2, -2.2, -2.4, -2.6, -2.8, -3, -3.2, -3.4, -3.6, -3.8, -4, -4.2, -4.4, -4.6, -4.8, -5, -5.2, -5.6, -8, -25, -75, -150, -300

**Supplementary Figure S1.** Example of multi-pool fitting of B_0_- and B_1_-corrected Z-spectra (B_1_ adjusted to 0.7 µT) acquired in the absence of fat (Phantom 1). The spectra are shown for vials containing increasing BSA concentrations (0–10% w/w) at a constant gelatin content (5% w/w). The fitting model included contributions from rNOE, amide, guanidino, and semisolid MT (ssMT) pools, which are expected to contribute to the Z-spectrum of BSA^1^ and gelatin^2^ in the low-power saturation regime^3,4^. This saturation regime was intentionally selected to preserve sensitivity to the BSA-related rNOE signal while also capturing amide- and guanidino-related contributions from the gelatine and BSA together as well as minimizing dilution by ssMT contributions.

**Supplementary Figure S2. CEST imaging results obtained in the fat-free phantom (phantom 1) at 3T.** **(A)** Representative averaged B_0_- and B_1_-corrected Z-spectra (B_1_ adjusted to 0.7 μT) acquired from vials 1–4 containing 5% (w/w) gelatin with increasing bovine serum albumin (BSA) concentrations. Compared with the corresponding Z-spectra acquired at 7T (Fig. 3), the Z-spectra acquired at 3T exhibit broader linewidths, which impedes reliable separation of the amide and guanidino contributions. In addition, spillover dilution due to the direct water saturation effects is also more pronounced at 3T. **(B)** Dependence of measured and B_1_-corrected MTR_Rex_ values for amide (3.5 ppm) and rNOE (−3.5 ppm) contrasts on BSA concentration (w/w). **(C)** Representative MTR_Rex_ maps for amide and rNOE contrasts.

**Supplementary Figure S3. CEST Z-spectra obtained in the fat-containing phantom (phantom 2) with and without fat-corrected normalization** **(A)** Representative Z-spectra averaged across vial’s ROIs. B_0_- and B_1_-corrected Z-spectra (B_1_ adjusted to 0.7 µT) acquired from vials 4–7 containing 5% (w/w) gelatin, 10% (w/w) bovine serum albumin (BSA), and increasing fat fraction (FF). **(B)** Representative fat signal-corrected Z-spectra with fat-corrected normalization applied as described in Zimmerman et al.^5^ averaged across vial’s ROIs. B_0_- and B_1_-corrected Z-spectra (B_1_ adjusted to 0.7 µT). Fat-corrected normalization enabled effective correction of the downfield part of the Z-spectrum (amide and guanidino signals) for FF values up to 35%, as previously described in^5^. This behavior is clearly reproduced in the phantom. For vials 5 and 6 (FF = 10% and 30%), the corrected direct water saturation (DWS) region around 0 ppm and the downfield part of the Z-spectrum (amide and guanidino signals) agree well both with each other and with the reference spectrum. In contrast, for the vial with FF = 50%, overcorrection of the downfield Z-spectrum is observed.

**Supplementary Table S1.** Representative T_1_, T_2_, fat fraction (FF), and pH values for a range of relevant tissues and organs at 7T

| **Tissue** | **T1 at 7T (ms)** | **T2 at 7T (ms)** | **FF / PDFF** | **pH** |
| --- | --- | --- | --- | --- |
| Current phantom | 1400 – 1600 | 7 – 70 | 0 – 50% | 7.1 – 7.3 |
| Brain GM | 1800 – 2100^6,7^ | 42 – 50^6,8,9^ | — | 7.00 – 7.10^10^ |
| Brain WM | 1100 – 1300^6,7^ | 27 – 34^6,8,9^ | — | 6.95 – 7.05^10,11^ |
| Fibroglandular breast tissue | 1500 – 2300^12,13^ | 35 – 64^12,13^ | 0 – 40%^14^ | — |
| Prostate | — | 63 – 100^15^ | 0 – 5%^16,17^ | 7.0 – 7.6^18^ |
| Liver | 1300 – 1400^19,20^ | ~15^19^ | 0 – 30%^21^ | 7.20 – 7.30^22^ |
| Spleen | ~1750^20^ | — | — | — |
| Kidney medulla | 2000 – 2600^20,23^ | ~130^23^ | 0 – 10%^24,25^ | 7.0^26^ |
| Kidney cortex | 1600 – 1900^20,23^ | ~110^23^ | 0 – 10%^24,25^ | 6.8^26^ |
| Cervical cord | ~1300 GM^27^,  ~1180 WM^27^ | — | — | — |
| Cartilage | — | ~60^28^ | — | ~6.6^29^ |
| Skeletal muscle | 1800 – 2000^20,30^ | ~22^30^ | 0 – 15%^31^ | 7.03 – 7.05^32^ |

**Supplementary Figure S4. CEST Z-spectra obtained in the phantom with different gelatin concentrations. (A)** Representative averaged across vial’s ROIs. B_0_- and B_1_-corrected Z-spectra (B_1_ adjusted to 0.7 µT) acquired from vials containing 2.2% (w/w) and increasing gelatin concentration. Small rNOE contribution can be seen, which was only possible to detect with low power saturation regime, used in current study. **(B)** Dependence of measured and B_1_-corrected MTR_Rex_ values for amide (3.5 ppm), guanidino (2.0 ppm), and rNOE (−3.5 ppm) contrasts on gelatin concentration (w/w), which also confirms presence of small rNOE signal.

**Reference list of Supporting Information**

1.	Smith AK, Ray KJ, Larkin JR, Craig M, Smith SA, Chappell MA. Does the magnetization transfer effect bias chemical exchange saturation transfer effects? Quantifying chemical exchange saturation transfer in the presence of magnetization transfer. *Magn Reson Med*. 2020;84(3):1359-1375. doi:10.1002/mrm.28212

2.	Liang Y, Bar-Shir A, Song X, Gilad AA, Walczak P, Bulte JWM. Label-Free Imaging of Gelatin-Containing Hydrogel Scaffolds. *Biomaterials*. 2015;42:144-150. doi:10.1016/j.biomaterials.2014.11.050

3.	Zaiss M, Jin T, Kim SG, Gochberg DF. Theory of chemical exchange saturation transfer MRI in the context of different magnetic fields. *NMR in Biomedicine*. 2022;35(11):e4789. doi:10.1002/nbm.4789

4.	Fabian MS, Rajput JR, Schüre JR, et al. Comprehensive 7 T CEST: A clinical MRI protocol covering multiple exchange rate regimes. *NMR in Biomedicine*. 2024;37(5):e5096. doi:10.1002/nbm.5096

5.	Zimmermann F, Korzowski A, Breitling J, et al. A novel normalization for amide proton transfer CEST MRI to correct for fat signal–induced artifacts: application to human breast cancer imaging. *Magnetic Resonance in Medicine*. 2020;83(3):920-934. doi:10.1002/mrm.27983

6.	Rooney WD, Johnson G, Li X, et al. Magnetic field and tissue dependencies of human brain longitudinal 1H2O relaxation in vivo. *Magnetic Resonance in Medicine*. 2007;57(2):308-318. doi:10.1002/mrm.21122

7.	Wright PJ, Mougin OE, Totman JJ, et al. Water proton T1 measurements in brain tissue at 7, 3, and 1.5T using IR-EPI, IR-TSE, and MPRAGE: results and optimization. *Magn Reson Mater Phy*. 2008;21(1):121. doi:10.1007/s10334-008-0104-8

8.	Seginer A, Schmidt R. Phase-based fast 3D high-resolution quantitative T2 MRI in 7 T human brain imaging. *Sci Rep*. 2022;12(1):14088. doi:10.1038/s41598-022-17607-z

9.	Uludağ K, Müller-Bierl B, Uğurbil K. An integrative model for neuronal activity-induced signal changes for gradient and spin echo functional imaging. *NeuroImage*. 2009;48(1):150-165. doi:10.1016/j.neuroimage.2009.05.051

10.	Cichocka M, Kozub J, Urbanik A. PH Measurements of the Brain Using Phosphorus Magnetic Resonance Spectroscopy (31PMRS) in Healthy Men – Comparison of Two Analysis Methods. *Pol J Radiol*. 2015;80:509-514. doi:10.12659/PJR.895178

11.	Korzowski A, Weinfurtner N, Mueller S, et al. Volumetric mapping of intra- and extracellular pH in the human brain using 31 P MRSI at 7T. *Magn Reson Med*. 2020;84(4):1707-1723. doi:10.1002/mrm.28255

12.	Bolan PJ, Snyder CJ, DelaBarre LJ, Bolinger L, Garwood M, Vaughan JT. Preliminary Experience with Breast 1H MRS at 7 Tesla.

13.	Brown R, Mcgorty K, Moy L, DeGregorio S, Sodickson DK, Wiggins GC. Sub-Millimeter Breast Imaging and Relaxivity Characterization at 7T.

14.	Borde T, Wu M, Ruschke S, et al. Assessing breast density using the chemical-shift encoding-based proton density fat fraction in 3-T MRI. *Eur Radiol*. 2023;33(6):3810-3818. doi:10.1007/s00330-022-09341-x

15.	Bonanno G, Jung B, Kreis R, et al. T2 Mapping of the Prostate at 7 Tesla: A Feasibility Study. In: Toronto, ON, Canada; :0525. doi:10.58530/2024/0525

16.	Zhao Y, Li G, Tian Z, et al. Quantitative Estimation of Iron and Fat Content in Prostate Cancer by Multiparametric MRI and Its Application in Optimizing D’Amico Score. *Journal of Magnetic Resonance Imaging*. 2025;61(5):2223-2233. doi:10.1002/jmri.29661

17.	Li G, Ding H, Tian Z, et al. Application of proton density fat fraction imaging in risk stratification of prostate cancer. *Transl Androl Urol*. 2024;13(9):1878-1890. doi:10.21037/tau-24-232

18.	Lagemaat MW, Vos EK, Maas MC, et al. Phosphorus magnetic resonance spectroscopic imaging at 7 T in patients with prostate cancer. *Invest Radiol*. 2014;49(5):363-372. doi:10.1097/RLI.0000000000000012

19.	Gajdošík M, Chmelík M, Just-Kukurová I, et al. In vivo relaxation behavior of liver compounds at 7 Tesla, measured by single-voxel proton MR spectroscopy. *J Magn Reson Imaging*. 2014;40(6):1365-1374. doi:10.1002/jmri.24489

20.	Bulanov P, Menshchikov P, Grimm JA, et al. Abdominal T1 relaxation times at 7T. *Magn Reson Mater Phy*. January 2026. doi:10.1007/s10334-025-01317-4

21.	Dag N, Sarici B, Igci G, Yagin FH, Yilmaz S, Kutlu R. Diagnostic Performance of Ultrasound-Based Liver Fat Quantification With Reference to Magnetic Resonance Imaging Proton Density Fat Fraction and Histology. *J Clin Ultrasound*. 2025;53(6):1265-1271. doi:10.1002/jcu.24010

22.	Rata M, Giles SL, deSouza NM, Leach MO, Payne GS. Comparison of three reference methods for the measurement of intracellular pH using^31^ P MRS in healthy volunteers and patients with lymphoma. *NMR in Biomedicine*. 2014;27(2):158-162. doi:10.1002/nbm.3047

23.	Li X, Bolan PJ, Ugurbil K, Metzger GJ. Measuring Renal Tissue Relaxation Times at 7T. *NMR Biomed*. 2015;28(1):63-69. doi:10.1002/nbm.3195

24.	Aydın H, Aydın H, Karaibrahimoğlu A, Afsar B. Evaluation of the parenchymal distribution of renal steatosis in chronic kidney disease using chemical shift magnetic resonance imaging. *Advances in Clinical and Experimental Medicine*. 2024;33(5):455-462. doi:10.17219/acem/166512

25.	Shen Y, Xie L, Chen X, et al. Renal fat fraction is significantly associated with the risk of chronic kidney disease in patients with type 2 diabetes. *Front Endocrinol (Lausanne)*. 2022;13:995028. doi:10.3389/fendo.2022.995028

26.	Wu Y, Zhou IY, Igarashi T, Longo DL, Aime S, Sun PZ. A generalized ratiometric chemical exchange saturation transfer (CEST) MRI approach for mapping renal pH using iopamidol. *Magnetic Resonance in Medicine*. 2018;79(3):1553-1558. doi:10.1002/mrm.26817

27.	Massire A, Taso M, Besson P, Guye M, Ranjeva JP, Callot V. High-resolution multi-parametric quantitative magnetic resonance imaging of the human cervical spinal cord at 7T. *NeuroImage*. 2016;143:58-69. doi:10.1016/j.neuroimage.2016.08.055

28.	Chang G, Xia D, Sherman O, et al. High resolution morphologic imaging and T2 mapping of cartilage at 7 Tesla: comparison of cartilage repair patients and healthy controls. *MAGMA*. 2013;26(6):539-548. doi:10.1007/s10334-013-0379-2

29.	High RA, Ji Y, Ma YJ, et al. In vivo assessment of extracellular pH of joint tissues using acidoCEST-UTE MRI. *Quant Imaging Med Surg*. 2019;9(10):1664-1673. doi:10.21037/qims.2019.08.11

30.	Marschar AM, Breithaupt M, Berger MC, Nagel AM. T1 and T2 relaxation times of the human calf at 7 Tesla.

31.	Schlaeger S, Inhuber S, Rohrmeier A, et al. Association of paraspinal muscle water-fat MRI-based measurements with isometric strength measurements. *Eur Radiol*. 2019;29(2):599-608. doi:10.1007/s00330-018-5631-8

32.	Lanza IR, Bhagra S, Nair KS, Port JD. Measurement of Human Skeletal Muscle Oxidative Capacity by 31P-MRS: A Cross-Validation with in vitro measurements. *J Magn Reson Imaging*. 2011;34(5):1143-1150. doi:10.1002/jmri.22733
